# Two Cases of Physical Therapy Focusing on the Meniscotibial Ligament and Its Associated Fat Pad for Medial Knee Pain in Knee Osteoarthritis

**DOI:** 10.7759/cureus.102356

**Published:** 2026-01-26

**Authors:** Yusuke Minamoto, Hyuga Takashima, Chihiro Honma, Koyuki Nakashima, Satoshi Chiba

**Affiliations:** 1 Department of Rehabilitation, Faculty of Health Sciences, Health Science University, Fujikawaguchiko, JPN; 2 Department of Rehabilitation, Chiba Child and Adult Orthopaedic Clinic, Chiba, JPN; 3 Department of Rehabilitation, Faculty of Health Sciences, Uekusa Gakuen University, Chiba, JPN

**Keywords:** fat pad, knee osteoarthritis, medial knee pain, medial meniscus, meniscotibial ligament, physical therapy, ultrasonography

## Abstract

Medial knee pain in knee osteoarthritis cannot always be explained solely by static structural abnormalities such as meniscal tears or osteophyte formation. Increasing attention has therefore been directed toward the dynamic behavior of peri-meniscal structures as potential contributors to symptom generation. This report describes two cases of knee osteoarthritis in which medial knee pain was localized near the tibial attachment of the medial meniscus (MM) despite minimal restriction of the knee range of motion and negative meniscal provocation tests. Dynamic ultrasonography revealed restricted MM displacement during tibial rotation in both cases, suggesting impaired adaptability of the meniscotibial ligament (MTL) and its underlying fat pad. Physical therapy focused on reducing tension in the MTL and facilitating physiological MM motion resulted in immediate and sustained pain reduction, accompanied by improvement in meniscal mobility on ultrasonographic assessment. These findings suggest that dynamic ultrasonographic evaluation combined with targeted physical therapy may represent a useful clinical approach for the assessment and management of medial knee pain in patients with knee osteoarthritis.

## Introduction

Medial knee pain in patients with knee osteoarthritis is influenced by multiple factors, often making clinical evaluation and pathophysiological interpretation challenging. One commonly implicated structure is the medial meniscus (MM), which plays an essential role in load distribution and joint stability through its hoop function [1]. Disruption of this function can lead to altered joint mechanics and various clinical symptoms.

However, structural abnormalities of the MM do not necessarily correlate with pain. Previous studies have shown that incidental meniscal findings on magnetic resonance imaging are frequently observed in asymptomatic middle-aged and elderly individuals, indicating that static structural changes alone cannot fully explain medial knee pain [2]. These findings highlight the importance of considering dynamic factors and the involvement of surrounding soft tissues when interpreting pain mechanisms in knee osteoarthritis.

The MM is anatomically integrated with surrounding capsuloligamentous structures. The medial collateral ligament (MCL) consists of superficial and deep layers, with the deep layer including the meniscofemoral ligament on the femoral side and the meniscotibial ligament (MTL) on the tibial side. Both ligaments attach directly to the MM and contribute to its physiological mobility during knee motion [3]. In knee osteoarthritis, tibial osteophyte formation has been reported to stretch the MTL laterally, resulting in MM extrusion and altered meniscal dynamics [4].

Recent anatomical studies have also identified a fat pad structure located beneath the MTL at the tibial margin of the medial joint space. This fat pad may function as a deformable interface that accommodates changes in ligament tension and facilitates smooth meniscal movement [5]. Dysfunction of this structure could therefore influence meniscal dynamics and contribute to pain generation.

In this report, we present two cases of knee osteoarthritis with medial knee pain localized near the tibial attachment of the MM. Despite minimal restriction of joint range of motion and negative meniscal stress tests, both cases demonstrated abnormal meniscal dynamics on ultrasonographic evaluation. Physical therapy focusing on the MTL and its underlying fat pad resulted in symptom improvement. The clinical course of these cases is presented, and the potential role of MTL-fat pad dysfunction in medial knee pain is discussed.

## Case presentation

Case 1

A man in his late 60s presented with medial knee pain and was diagnosed with right knee osteoarthritis (Kellgren-Lawrence grade II). His chief complaint was medial knee pain during deep knee flexion, particularly when sitting in a cross-legged position and when attempting to squat, which had persisted for several months. He reported no difficulty with walking or stair climbing.

Physical examination revealed localized tenderness at the tibial aspect of the medial joint line. The semimembranosus tendon, which courses adjacent to and inserts near the medial joint line, did not demonstrate focal tenderness, including its anterior and direct arms. Meniscal and ligamentous stress tests were performed and did not indicate a clear primary pathology of the meniscus or MCL.

The patient ambulated independently with a symmetrical gait pattern. Lower extremity muscle strength was preserved, and knee range of motion demonstrated only mild limitation in extension and deep flexion.

**Figure 1:**
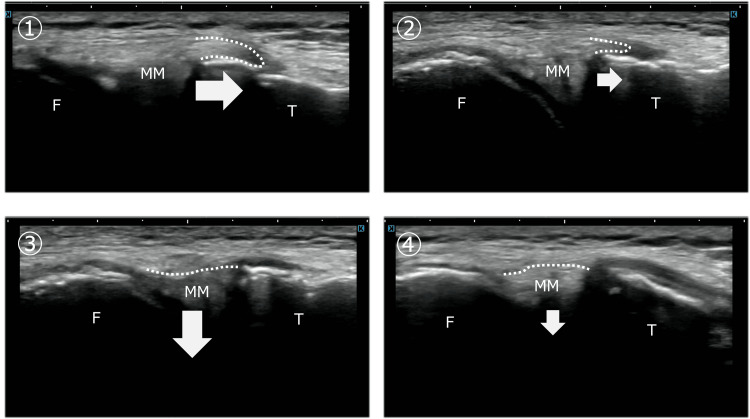
Dynamic movement of the medial meniscus during tibial rotation: comparison between the unaffected and affected sides on ultrasonography Ultrasonography was performed with the knee in slight flexion while tibial internal and external rotation was applied to assess medial meniscus (MM) movement associated with changes in medial collateral ligament (MCL) tension. Panels ① and ② represent tibial internal rotation on the unaffected and affected sides, respectively. On the unaffected side, the MM moved smoothly toward the extracapsular side during internal rotation, whereas on the affected side, the magnitude of MM movement was reduced. Panels ③ and ④ represent tibial external rotation on the unaffected and affected sides, respectively. On the unaffected side, the MM entered the medial joint space sufficiently during external rotation; however, on the affected side, inward excursion of the MM was limited. These findings indicate restricted MM dynamics on the affected side during both internal and external tibial rotation. F, femur; T, tibia

Dynamic ultrasonography was performed with the knee maintained in slight flexion, while controlled tibial internal and external rotation was applied to assess MM displacement in relation to tension changes of the MCL and MTL. On the contralateral side, tibial external rotation increased MCL tension and guided the MM into the joint space, whereas tibial internal rotation relaxed the MCL and MTL, allowing the MM to move outside the joint line and traverse the tibial margin. In contrast, on the affected side, shape changes of the MCL and MTL were minimal during tibial rotation, and MM displacement was markedly reduced. The MM failed to traverse the tibial margin during tibial internal rotation (Figure 1).

Treatment and Outcome

**Figure 2:**
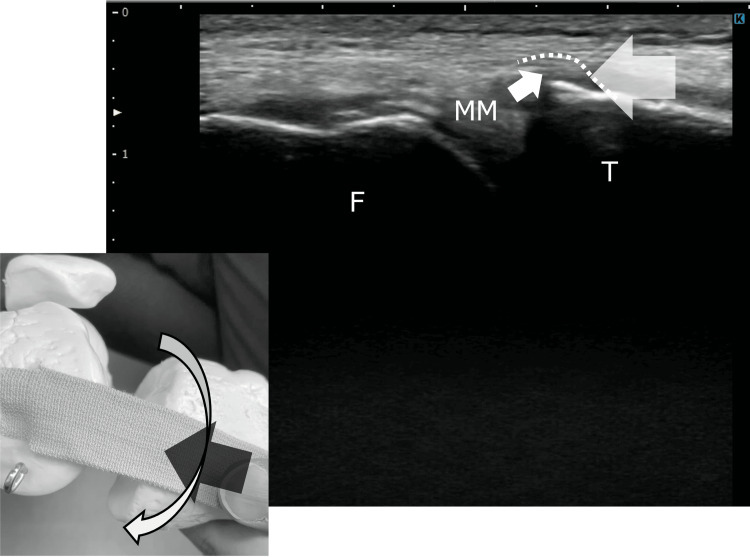
Conceptual illustration of the manual intervention based on ultrasonographic findings This figure schematically illustrates the manual technique designed to improve medial meniscus (MM) mobility, based on dynamic findings obtained from ultrasonography. Approximating the distal portion of the medial collateral ligament (MCL) toward the proximal direction is intended to induce shape changes in the meniscotibial ligament (MTL) and the underlying fat pad, thereby creating a potential space beneath the ligament. Under this condition, tibial rotation is expected to facilitate smoother movement of the MM into the created space. Repetition of this maneuver was hypothesized to promote improved MM mobility. F: femur; T: tibia; MM: medial meniscus

Treatment focused on restoring physiological MM motion by improving mobility of the MTL and the fat pad beneath it. Manual techniques were applied to approximate the MCL from distal to proximal, thereby reducing tension in the MTL. This intervention was intended to modify the mechanical environment beneath the MTL by improving the adaptability of the underlying fat pad, thereby allowing smoother coordinated motion of the MTL and MM and reducing sustained focal pressure at the tibial margin. While maintaining this position, controlled tibial internal rotation was performed to encourage the MM to move into the space formed beneath the ligament (Figure 2).

**Figure 3:**
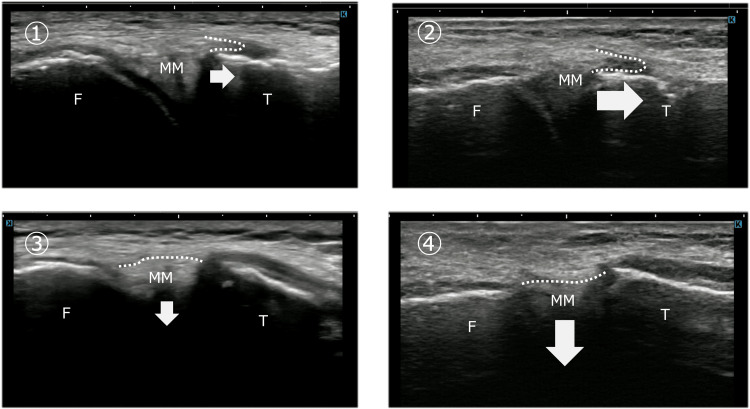
Improvement in medial meniscus mobility after physical therapy in Case 1 Ultrasonographic images demonstrating changes in medial meniscus (MM) dynamics after physical therapy in Case 1. Panels ①→② represent tibial internal rotation, and panels ③→④ represent tibial external rotation. After intervention, the MM demonstrated improved excursion toward both the tibial side and the joint space, indicating restored mobility. These changes were accompanied by resolution of pain during deep knee flexion and cross-legged sitting. F: femur; T: tibia; MM: medial meniscus

Pain decreased immediately after treatment, and ultrasonography confirmed improved MM mobility comparable to the contralateral side (Figure 3). Treatment was performed once weekly for two months, resulting in complete resolution of pain during cross-legged sitting and squatting.

Case 2

A woman in her late 60s presented with medial knee pain and was diagnosed with bilateral knee osteoarthritis (Kellgren-Lawrence grade II), with predominant symptoms in the right knee. Her chief complaint was pain during sit-to-stand movements and stair ascent, while level walking caused minimal discomfort. The symptoms had initially developed approximately seven to eight years earlier, with gradual progression over time.

Physical examination revealed tenderness localized to the tibial aspect of the medial joint line. Knee range of motion was 140° of flexion bilaterally, with extension of -5° on the right and 0° on the left, indicating mild limitation in extension on the symptomatic side. Lower extremity muscle strength was symmetrical, and resisted movements were pain-free. Pain was noted during functional movements involving knee extension under load, particularly during transitional tasks.

Pain was reproducibly elicited during the phase from seat-off to early knee extension during the sit-to-stand movement, particularly after prolonged sitting. Initial treatment focused on improving muscle flexibility and joint range of motion. Although knee flexion improved to 150° and extension to 0°, pain during the sit-to-stand movement persisted.

**Figure 4:**
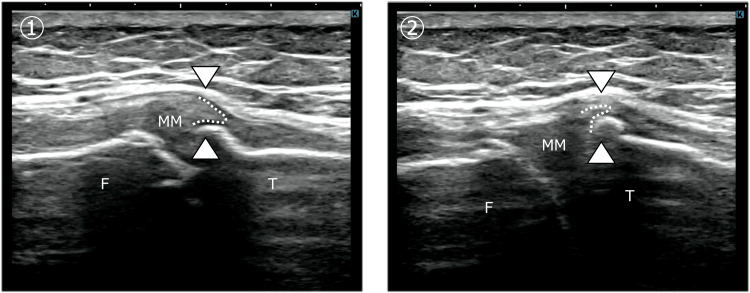
Restricted medial meniscus mobility due to osteophyte impingement in Case 2 Ultrasonographic images of the affected knee in Case 2 obtained at a slightly flexed position, demonstrating restricted medial meniscus (MM) dynamics during tibial rotation. Panel ① shows tibial internal rotation, and panel ② shows tibial external rotation. In both conditions, the tibial edge of the medial meniscus was impinged by a medial tibial osteophyte (arrowheads), preventing smooth excursion into and out of the joint space. This mechanical interference corresponded to the reproduction of movement-related pain. F: femur; T: tibia; MM: medial meniscus

Dynamic ultrasonography revealed limited MM displacement during both tibial internal and external rotation on the symptomatic side. Mechanical interference between the MM and a tibial osteophyte was observed, preventing smooth meniscal movement into and out of the joint space (Figure 4).

Treatment and Outcome

Treatment focused on creating space beneath the MTL using manual techniques consistent with those applied in Case 1. Manual approximation of the MCL from distal to proximal was performed, followed by controlled tibial rotation to facilitate smooth MM movement (Figure 2).

**Figure 5:**
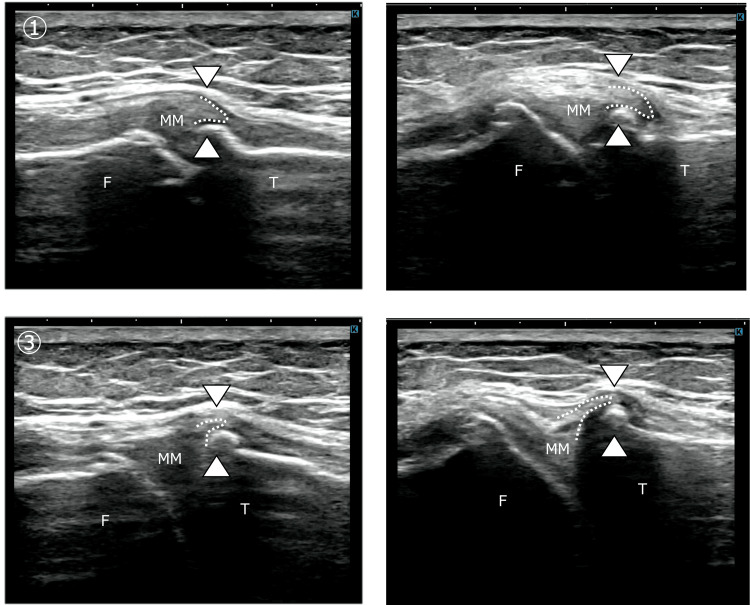
Improvement of medial meniscus excursion after physical therapy in Case 2 Ultrasonographic images of the affected knee in Case 2 obtained after physical therapy, demonstrating improved medial meniscus (MM) dynamics during tibial rotation. Panels ①→② represent tibial internal rotation, and panels ③→④ represent tibial external rotation. During tibial internal rotation, the MM, which had previously been mechanically restricted by osteophyte impingement, demonstrated increased inferior displacement along the tibial margin, allowing it to pass beyond the mid-slope of the osteophyte after intervention (①→②). During tibial external rotation, the MM showed smooth displacement from the tibial margin toward the joint space, indicating restoration of physiological meniscal mobility (③→④). After the intervention, neither snapping phenomena nor mechanical impingement between the MM and the osteophyte were observed. F: femur; T: tibia; MM: medial meniscus

Pain during sit-to-stand movements decreased immediately from a numerical rating scale score of 5 to 2. With continued weekly treatment, the snapping phenomenon gradually diminished and resolved by week 12 (Figure 5). Ultimately, pain during standing up and stair climbing decreased to a numerical rating scale score of 1, with no limitation in activities of daily living.

## Discussion

Medial knee pain in knee osteoarthritis is often difficult to explain solely by static structural abnormalities such as meniscal tears or osteophyte formation. The MM plays an important role in load distribution through its hoop function [1]. However, Englund et al. reported that incidental meniscal abnormalities on imaging do not necessarily correlate with pain, indicating that dynamic factors and surrounding soft tissues should be considered when interpreting pain mechanisms [2].

The dynamics of the MM are strongly influenced by surrounding capsuloligamentous structures, particularly in pathological conditions. The deep layer of the MCL includes the MTL, which attaches to the tibial side of the MM and contributes to its physiological mobility [3]. In knee osteoarthritis, tibial osteophyte formation has been shown to stretch the MTL laterally, contributing to MM extrusion [4]. In the present cases, restoring the flexibility of the fat pad beneath the MTL likely reduced persistent mechanical constraint on the MM, enabling more physiological meniscal motion during subsequent weight-bearing activities such as sit-to-stand and squatting.

Beneath the MTL, a fat pad structure has been identified at the tibial margin of the medial joint space [5]. Morphological characteristics of the MCL and its deep components, including the MTL, have been shown to influence ligament behavior during knee motion [6]. This fat pad is thought to function as a deformable interface that passively adapts to changes in ligament tension and facilitates smooth meniscal movement. Fat pads are not merely space-filling tissues but are increasingly recognized as functional structures that contribute to load dispersion and gliding mechanics within the knee joint [7].

In both cases presented, ultrasonography demonstrated restricted MM mobility during tibial rotation, despite minimal limitations in joint range of motion and negative meniscal or ligamentous stress tests. Pain was reproduced during movements requiring coordinated stretching and relaxation of the MTL. These findings suggest that reduced deformability of the fat pad beneath the MTL may have limited ligament adaptability, resulting in impaired MM dynamics and localized mechanical stress between the MM and tibial osteophytes. Chronic mechanical stress has been reported to induce fibrosis and reduced viscoelasticity of adipose tissue, which may limit its ability to adapt to surrounding tissue motion [8].

After physical therapy aimed at reducing MTL tension and facilitating space formation beneath the ligament, MM mobility improved immediately, accompanied by pain reduction. In Case 1, symptoms during deep flexion resolved, whereas in Case 2, pain during standing and stair climbing gradually disappeared. Although the pain-provoking movements differed between cases, both involved rotational knee motion, supporting a shared pathophysiological mechanism related to MTL-fat pad dysfunction.

This report has limitations, as changes in the physical properties of the sub-MTL fat pad were not directly assessed. However, the dynamic ultrasonographic findings and immediate clinical response suggest that dysfunction of the MTL and its underlying fat pad may contribute to medial knee pain in knee osteoarthritis.

Although the present report is limited to two cases, these findings indicate that dynamic assessment of peri-meniscal structures, including the MTL and its underlying fat pad, may be clinically relevant in selected patients with medial knee pain that cannot be adequately explained by static imaging findings alone. Ultrasonography-based dynamic evaluation may help identify abnormal meniscal behavior during symptom-provoking movements and guide targeted physical therapy interventions. Nevertheless, given the limited sample size and observational nature of this report, the generalizability of these findings is restricted, and further studies involving larger cohorts are required to clarify the clinical applicability of this approach.

## Conclusions

These cases suggest that dysfunction of the MTL and its underlying fat pad may contribute to medial knee pain in knee osteoarthritis by impairing physiological MM motion. Dynamic ultrasonographic assessment combined with targeted physical therapy focusing on these peri-meniscal structures may provide a useful clinical approach in selected patients whose symptoms cannot be fully explained by static imaging findings alone.

## References

[1] Toyama H (2020). Special feature: meniscus—save the meniscus I. Fundamentals of the meniscus: biomechanics of the meniscus [Article in Japanese]. Orthop Surg Traumatol.

[2] Englund M, Guermazi A, Gale D, Hunter DJ, Aliabadi P, Clancy M, Felson DT (2008). Incidental meniscal findings on knee MRI in middle-aged and elderly persons. N Engl J Med.

[3] Śmigielski R, Becker R, Zdanowicz U, Ciszek B (2015). Medial meniscus anatomy-from basic science to treatment. Knee Surg Sports Traumatol Arthrosc.

[4] Hada S, Ishijima M, Kaneko H (2017). Association of medial meniscal extrusion with medial tibial osteophyte distance detected by T2 mapping MRI in patients with early-stage knee osteoarthritis. Arthritis Res Ther.

[5] Cavaignac E, Sylvie R, Teulières M, Fernandez A, Frosch KH, Gomez-Brouchet A, Sonnery-Cottet B (2021). What is the relationship between the distal semimembranosus tendon and the medial meniscus? A gross and microscopic analysis from the SANTI Study Group. Am J Sports Med.

[6] Liu F, Yue B, Gadikota HR (2010). Morphology of the medial collateral ligament of the knee. J Orthop Surg Res.

[7] Macchi V, Stocco E, Stecco C, Belluzzi E, Favero M, Porzionato A, De Caro R (2018). The infrapatellar fat pad and the synovial membrane: an anatomo-functional unit. J Anat.

[8] Dragoo JL, Johnson C, McConnell J (2012). Evaluation and treatment of disorders of the infrapatellar fat pad. Sports Med.

